# How Amdahl’s Law limits the performance of large artificial neural networks: why the functionality of full-scale brain simulation on processor-based simulators is limited

**DOI:** 10.1186/s40708-019-0097-2

**Published:** 2019-04-11

**Authors:** János Végh

**Affiliations:** Kalimános BT, Komlóssy u 26, Debrecen, 4032 Hungary

**Keywords:** Amdahl’s Law, Neural networks, Performance limitation, Clock-driven electronic circuit, Supercomputing, Parallelization

## Abstract

With both knowing more and more details about how neurons and complex neural networks work and having serious demand for making performable huge artificial networks, more and more efforts are devoted to build both hardware and/or software simulators and supercomputers targeting artificial intelligence applications, demanding an exponentially increasing amount of computing capacity. However, the inherently parallel operation of the neural networks is mostly simulated deploying inherently sequential (or in the best case: sequential–parallel) computing elements. The paper shows that neural network simulators, (both software and hardware ones), akin to all other sequential–parallel computing systems, have computing performance limitation due to deploying clock-driven electronic circuits, the 70-year old computing paradigm and Amdahl’s Law about parallelized computing systems. The findings explain the limitations/saturation experienced in former studies.

## Introduction

Although von Neumann in his classic publication [1, 2] targeted creating *computing organ*s, developers had to consider the that-time technological possibilities and the urgent need to automate numeric calculations. In this way, the primary goal was to *compute* and all efforts (at that time and the next seven decades) were devoted to develop the device *computer*. The sound success of that automated computing and the growing scientific, industrial, military, etc. needs targeted to increase the *single-processor performance*, although more than five decades ago it was already recognized [3] that when utilizing that computing principle one faces serious performance limitations. The today’s goal to simulate operation of even a relatively small-scale (compared to human brain) neural network in real-time faces serious difficulties and leads to serious performance issues [4]. In the light of that biological neurons work in the millisecond range, the silicon neurons could work in the nanosecond range and that both software-only neural network simulator programs running on supercomputers built from general-purpose processors and special-purpose hardware brain-simulator utilize million(s) of high-performance processors produce such a low performance, the question raises: *where do we loose several orders of magnitude of computing performance*?

One obvious candidate for loosing performance is the general-purpose processor itself [5]: its performance is awfully low compared to implementation in Application Specific Integrated Circuits. However, it cannot be responsible alone for the full loss. The development of single-processor performance has stalled [6] and only marginal development of single-processor performance can be expected. The computing needs renewing [7, 8] because of many reasons. One of them is that the many forms of increasing the performance through various kinds of parallelization [9] is a short dead-end street: the computing efficiency exponentially decreases as the number of processors increases [10], the today’s supercomputers are stretched to their limits [11] in the case of large-scale systems. A slowdown or stalling of the growth of their performance is expected according to the general experiences [12] and is really experienced among others in the field of supercomputing [13]. However, similarly to most of the basic limitations of computing, even the limitations are limited [14].

The goal of the paper is to show that akin to supercomputers the computing performance of the processor-based brain simulators is also limited. As discussed below, utilizing (mainly: thinking in) clocked electronic circuits, the 70-year-old single-processor approach and Amdahl’s Law together result in that only marginal developments in the performance of neuromorphic simulations can be expected. The question only is *how far brain simulation is from its limitations*? And of course, *whether those limitations are limited*, too?

The paper first interprets *Amdahl’s Law* for any kind of parallelized activity in Sect. 2 and shows in Sect. 3 that utilizing clock signals in the electronic circuits leads to an inherent limitation of performance. Section 4 shows how in supercomputing this inherent limitation bounds the achievable computing performance. Although brain simulation seems to be unrelated to the sequential/parallel computing world, in Sect. 5 it is shown that the technical implementation introduces a hidden clock signal into neural computing. This section also reveals the origins of the saturation effects experienced in former studies. Finally, in Sect. 6, some ideas are proposed for using some alternative approach to get closer to full-power brain simulations.

## Amdahl’s Law of parallelization

### Amdahl’s idea

*Amdahl’s Law is one of the few, fundamental laws of computing* [15], although sometimes it is partly or completely misinterpreted or abused [15–17]. A general misconception (introduced by successors of Amdahl) is to assume that Amdahl’s law is valid for software only. Actually,* Amdahl’s law is valid for any partly parallelizable activity (including computer unrelated ones) and the non-parallelizable fragment shall be given as the ratio of the time spent with non-parallelizable activity to the total time*. Notice that Amdahl speaks about partly parallelizable activities in simple parallelized systems, unlike complex sequential–parallel systems with variable degree of parallelism [18] or the brain operation. That is, Amdahl’s law can be directly applied only to such simple systems, but the idea itself can be utilized to study even the mentioned complex systems.

### Deriving the effective parallelization

Successors of Amdahl expressed *Amdahl’s law* with the formula

1 $$\begin{aligned} S^{-1}=(1-\alpha ) +\alpha /k \end{aligned}$$

where *k* is the number of parallelized activities, $\alpha$ is the ratio of the parallelizable part within the total activity, *S* is the measurable speedup. The assumption can be visualized that (in a conventional computing system assuming many processors) in $\alpha$ fraction of the running time the processors are executing parallelized code, in ($1-\alpha$) fraction they are waiting (all but one), or making non-payload activity. That is $\alpha$ describes how much, in average, processors are utilized, or how effective (at the level of the computing system) the parallelization is.

For a system under test, where $\alpha$ is not *a priory* known, one can derive from the measurable speedup *S* an *effective parallelization* factor [19] as

2 $$\begin{aligned} \alpha _{\mathrm{eff}} = \frac{k}{k-1}\frac{S-1}{S} \end{aligned}$$

Obviously, this is not more than $\alpha$ expressed in terms of *S* and *k* from Eq. (1). For the classical case, $\alpha = \alpha _{\mathrm{eff}}$; which simply means that in the *ideal* case the actually measurable effective parallelization achieves the theoretically possible one. In other words, $\alpha$ describes a system the *architecture* of which is completely known, while $\alpha _{\mathrm{eff}}$ characterizes the *performance*, which describes both the complex architecture and the actual conditions. It was also demonstrated [19] that $\alpha _{\mathrm{eff}}$ can be successfully utilized to describe parallelized behavior from software (SW) load balancing through measuring efficacy of the on-chip communication inside a hardware (HW) unit to characterizing performance of clouds.

The value $\alpha _{\mathrm{eff}}$ can also be used to refer back to Amdahl’s classical assumption even in the realistic case when the parallelized chunks have different lengths and the overhead to organize parallelization is not negligible. The speedup *S* can be measured and $\alpha _{\mathrm{eff}}$ can be utilized to characterize the measurement setup and conditions, how much from the theoretically possible maximum parallelization is realized. Numerically ($1-\alpha _{\mathrm{eff}}$) equals with the *f* value, established theoretically [20].

The distinguished constituent in Amdahl’s classic analysis is the parallelizable fraction $\alpha$, all the rest (including wait time, non-payload activity, etc.) goes into the “sequential-only” fraction. When using several processors, one of them makes the sequential calculation, the others are waiting (use the same amount of time). So, when calculating the speedup, one calculates

3 $$\begin{aligned} S=\frac{(1-\alpha )+\alpha }{(1-\alpha )+\alpha /k} =\frac{k}{k(1-\alpha )+\alpha } \end{aligned}$$

hence the efficiency is (see also Fig. 2).

4 $$\begin{aligned} E = \frac{S}{k}=\frac{1}{k(1-\alpha )+\alpha } \end{aligned}$$

This explains the behavior of diagram $\frac{S}{k}$ in function of *k* experienced in practice: the more processors, the lower efficiency. At this point, one can notice that $\frac{1}{E}$ is a linear function of number of processors, and its slope equals to $(1-\alpha )$. Equation (4) also underlines the importance of the single-processor performance: the lower is the number of the processors used in the parallel system having the expected performance, the higher can be the efficacy of the system.

Notice also, that through using Eq. (4), the efficiency $\frac{S}{k}$ (which is given for supercomputers as $\frac{R_{\mathrm{Max}}}{R_{\mathrm{Peak}}}$) can be equally good for describing the efficiency of parallelization of a setup, provided that the number of processors is also known. From Eq. (4)

5 $$\begin{aligned} \alpha _{E,k} = \frac{E k -1}{E (k-1)} \end{aligned}$$

If the parallelization is well-organized (load balanced, small overhead, right number of processors), $\alpha _{\mathrm{eff}}$ is close to unity, so tendencies can be better displayed through using $(1-\alpha _{\mathrm{eff}})$ in the diagrams.

The importance of this practical term $\alpha _{\mathrm{eff}}$ is underlined by that it can be interpreted and utilized in many different areas [19] and the achievable speedup (the maximum achievable performance gain when using infinitely large number of processors) can easily be derived from Eq. (1) as

6 $$\begin{aligned} G=\frac{1}{(1-\alpha _{\mathrm{eff}})} \end{aligned}$$

Provided that the value of $\alpha _{\mathrm{eff}}$ does not depend on the number of the processors, for a homogenous system the total payload performance is

7 $$\begin{aligned} P_{\mathrm{total\,payload}}=G\cdot P_{\mathrm{single\,processor}} \end{aligned}$$

i.e., *the total payload performance can be increased by increasing the performance gain or increasing the single-processor performance, or both*. Notice, however, that increasing the single-processor performance through accelerators also has its drawbacks and limitations [21], and that the performance gain and the single-processor performance are players of the same rank in defining the payload performance.

Notice the special role of the non-parallelizable activities: independently of their origin, they are summed up as ‘sequential-only’ contribution and degrade considerably the payload performance. In systems comprising parallelized sequential processes actions like communication (including also MPI), synchronization, accessing shared resources, etc. [22–25] all contribute to the sequential-only part. Their effect becomes more and more drastic as the number of the processors increases. One must take care, however, *how* the communication is implemented. A nice example is shown in [26], how direct core to core (in other words: direct thread to thread) communication can enhance parallelism in large-scale systems.

## The inherent limit of parallelization

In the systems implemented in single-processor approach (SPA) [3] as parallelized sequential systems, the life begins in one such sequential subsystem. For example, in large parallelized applications running on general-purpose supercomputers, initially and finally only one thread exists, i.e., the minimal absolutely necessary non-parallelizable activity is to fork the other threads and join them again. With the present technology, no such actions can be shorter than one processor clock period.^1^ That is, the absolute minimum value of the non-parallelizable fraction will be given as the ratio of the time of the two clock periods to the total execution time. The latter time is a free parameter in describing the efficiency, i.e., value of the effective parallelization $\alpha _{\mathrm{eff}}$ *also depends on the total benchmarking time* (and so does the achievable parallelization gain, too).

This dependence is of course well known for supercomputer scientists: for measuring the efficiency with better accuracy (and also for producing better $\alpha _{\mathrm{eff}}$ values) hours of execution times are used in practice. For example, in the case of benchmarking the supercomputer ${ \it Taihulight}$ [27] 13,298 s benchmark runtime was used; on the 1.45 GHz processors it means $2*10^{13}$ clock periods. This means that (at such benchmarking time) the inherent limit of $(1-\alpha _{\mathrm{eff}})$ is $10^{-13}$ (or equivalently the achievable performance gain is $10^{13}$). If the fork/join is executed by the operating system (OS) as usual, because of the needed context switchings $2*10^{4}$ [28] clock cycles are needed rather than the 2 clock cycles considered in the idealistic case, i.e., *the derived values are correspondingly by 4 orders of magnitude different; that is the performance gain cannot be above* $10^9$. When making only 10 s long measurements, the smaller denominator results in $10^3$ times worse $(1-\alpha _{\mathrm{eff}})$ and performance gain values. In the following for simplicity 1.00 GHz processors (i.e., 1 ns clock cycle time) will be assumed.

There are other similar limitations in consequence of the physical implementation. The supercomputers, for example, are also distributed systems. In a stadium-sized supercomputer, the distance between processors (cable length) about 100 m can be assumed. The net signal round trip time is ca. $10^{-6}$ s, or $10^{3}$ clock periods, i.e., in the case of a finite-sized supercomputer the performance gain cannot be above $10^{10}$ (or $10^{6}$ if context switching also needed). The presently available network interfaces have 100...200 ns latency times, and sending a message between processors takes time in the same order of magnitude. This also means that *making better interconnection is not really a bottleneck in enhancing performance*, at least when measured using the benchmark High-Performance Linpack (HPL). This statement is underpinned also by statistical considerations [21].

Taking the (maybe optimistic) value $2*10^{3}$ clock periods for the signal propagation time, the value of the effective parallelization $(1-\alpha _{\mathrm{eff}})$ will be at best in the range of $10^{-10}$, only because of the physical size of the supercomputer. This also means that the expectations against the absolute performance of supercomputers are excessive: assuming a 100 Gflop/s processor, the achievable absolute *nominal* performance (see Eq. (6)) is $10^{11}$*$10^{10}\,{\mathrm{flop/s}}$, i.e., 1000 EFlops. To implement this, around $10^9$ processors are required. One can assume that the value of $(1-\alpha _{\mathrm{eff}})$ will be^2^ around of the value $10^{-7}$. With those very optimistic assumptions (see Eq. 4), the *payload* performance for benchmark HPL will be less than 10 Eflops, and for the real-life applications of class of the benchmark High-Performance Conjugate Gradients (HPCG), it will be surely below 0.01 EFlops, i.e., lower than the payload performance of the present TOP1-3 supercomputers.

These predictions enable to assume that the presently achieved value of $(1-\alpha _{\mathrm{eff}})$ persists also for roughly hundred times more cores. However, another major issue arises from the computing principle single-processor approach (SPA): only one computer at a time can be addressed by the first one. As a consequence, minimum as many clock cycles are to be used for organizing the parallel work as many addressing steps required. Basically, this number equals to the number of cores in the supercomputer, i.e., the addressing in the TOP10 positions typically needs clock cycles in the order of $5*10^{5}$...$10^{7}$; degrading the value of $(1-\alpha _{\mathrm{eff}})$ into the range $10^{-6}$...$2*10^{-5}$. Two tricks may be used to mitigate the number of the addressing steps: either the cores are organized into *cluster*s as many supercomputer builders do, or at the other end the processor itself can take over the responsibility of addressing its cores [29]. Depending on the actual construction, the reducing factor of clustering of those types can be in the range $10^{2}$...$5*10^{4}$, i.e., the resulting value of $(1-\alpha _{\mathrm{eff}})$ is expected to be around $10^{-7}$. Notice that utilizing “cooperative computing” [29] enhances further the value of $(1-\alpha _{\mathrm{eff}})$, but it means already utilizing a (slightly) different computing paradigm: the cores have a kind of direct connection and can communicate with the exclusion of the main memory.

An operating system must also be used, for protection and convenience. If one considers context switching with its consumed $2*10^4$ cycles [28], the absolute limit is cca. $5*10^{-8}$, on a zero-sized supercomputer. This value is somewhat better than the limiting value derived above, but it is close to that value and surely represents a considerable contribution. This is why ${\it Taihulight}$ runs the actual computations in kernel mode [29].

Because all of this, in the name of the company PEZY^3^, the last two letters are surely obsolete. Also, no Zettaflops supercomputers will be delivered for science and military [30]. It looks like that in the feasibility studies, an analysis on whether this inherent performance bound exists is done neither in USA [31] nor in EU [32] nor in Japan [33] nor in China [13].

As discussed above, some limitations follow immediately from the physical implementation and the computing paradigm; it depends on the actual conditions, which of them will dominate. *It is crucial to understand that the decreasing efficiency (see Eq.* (4)) *is coming from the computing paradigm itself rather than from some kind of engineering imperfectness. This inherent limitation cannot be mitigated without changing the computing/implementation principle.*

## The inherent limits of supercomputing

The considerations on supercomputing result in a (by intention oversimplified) parametrized model that qualitatively describes the behavior of the supercomputers during its 26 years of history, and the parameters can be estimated from the public, rigorously controlled, accurate database [34]. The model [10, 35] not only describes the performance of the presently existing supercomputers, but also predicts their future achievable performance.

Although not explicitly dealt with here, notice that the data exchange between the first thread and the other ones also contribute to the non-parallelizable fraction and typically uses system calls, for details see [23–25]. Actually, we may have communicating serial processes, which does not improve the effective parallelism at all [22]. Essentially, this is why supercomputers have a “benchmark efficiency” (measured by HPL) and a “practical efficiency” (measured by benchmark HPCG), the latter of which is 100...1000 times lower than the former one. For details see [10]. Recall that artificial neurons make intensive data exchange among each other, so they surely belong to the HPCG class of applications, although utilizing “far” and “near” memories can change their behavior drastically.

**Fig. 1:**
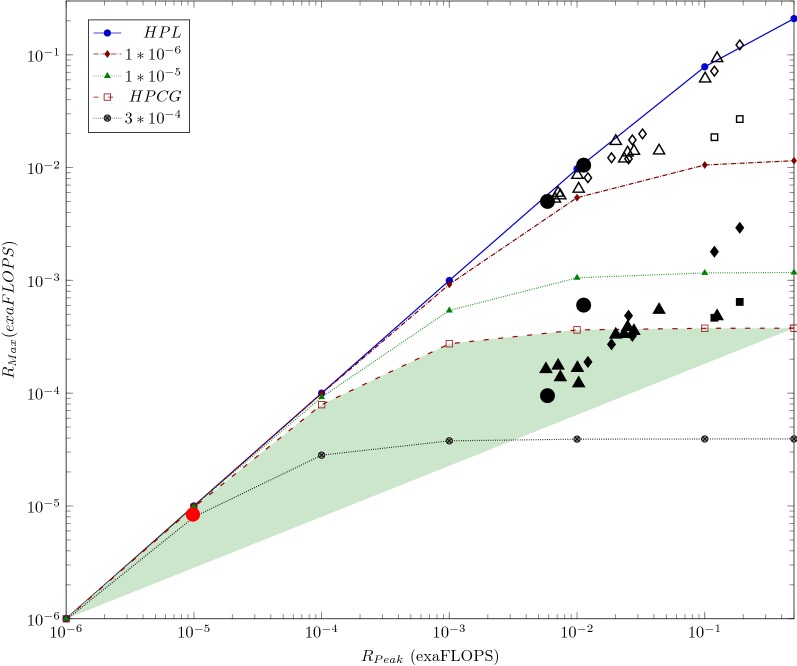
The $R_{\mathrm{Max}}$ payload performance in function of the nominal performance $R_{\mathrm{Peak}}$, at different $(1-\alpha _{\mathrm{eff}})$ values. The figures display the measured values derived using HPL (empty marks) and HPCG (filled marks) benchmarks, for the TOP15 supercomputers. The computing performance of AI applications may be similar to the diagram line marked by HPCG. The diagram lines marked as HPL and HPCG correspond to the behavior of supercomputer ${\it Taihulight}$ at $(1-\alpha _{\mathrm{eff}})$ values $3.3*10^{-8}$ and $2.4*10^{-5}$, respectively. The uncorrected values of the new supercomputers ${{ Summit}}$ and ${ Sierra}$ are shown as diamonds, and the same values corrected for single-processor performance are shown as rectangles. The black dots mark the performance data of supercomputers ${ JUQUEEN}$ and *K* as of 2014 June, for HPL and HPCG benchmarks, respectively. The red dot denotes the performance value of the system used by [4]. The saturation effect can be observed for both HPL and HPCG benchmarks. The shaded area only highlights the nonlinearity

Figure 1 depicts how the *payload performance* of the benchmarks HPL and HPCG depend on the *nominal performance* of the general-purpose supercomputers. The diagram lines referring to different $(1-\alpha _{\mathrm{eff}})$ values are calculated from the model [10], the marks are measured for the different TOP15 supercomputers (as of Nov 2018). The empty marks refer to the HPL and the filled ones to the HPCG benchmarks. For more legends and explanations see [10]. Notice that the simulators of artificial neural networks show up a behavior quite close to the HPCG class applications, see below.

## The inherent limits of neural network simulation

Of course, brain simulation as such has nothing to do with clock-driven sequential-parallel processing. However, brain simulators (the SW ones and most of the HW ones) run on such computing systems (are built from such components) and inherit the limitations valid for those systems. As discussed above, Eq. (7) defines the achievable payload performance of a parallelized computing system, and also that the performance gain is limited by the clock frequency.

The living “architectures” have no central clock signal, so the simulations should not have one, too. Despite this, from technology reasons, all simulations utilize a “time grid”, commonly with 1 ms integration time. This grid time is used to put the free running neurons back to the biological time scale, i.e., they actually act as a clock signal: stimulating of the next calculation step can only start when this clock signal arrives. This is absolutely analogous with the goal of introducing clock signal for executing the machine instructions: the processor, even when it is idle, cannot begin the execution of the next machine instruction until the clock signal arrives. The artificial neural networks constructed in this way can be considered as consisting from nodes having rather complex internal operation (implementing exceptionally high computing performance) and concerted by the clock signal which is technically required to enable performing the concerted integration.

**Fig. 2:**
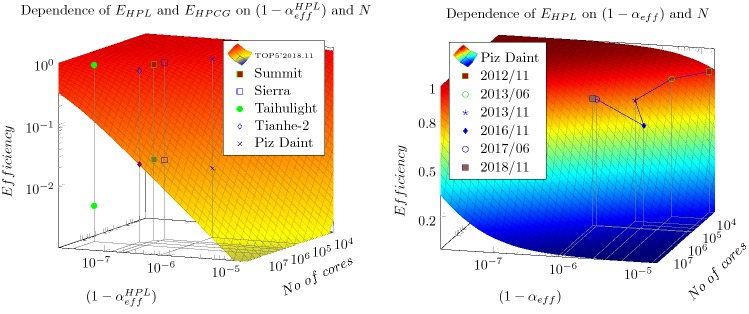
The dependence of the efficiency E on the number of the cores and the value of $(1-\alpha _{\mathrm{eff}}^{X})$ , as described by Equ. (4). For comparison both the corresponding measured $(1-\alpha _{\mathrm{eff}}^{X})$ values of the first five computers (as of 2018 November) are also displayed, as measured by the benchmarks HPL and HPCG

If the artificially introduced "biological clock cycle" is not considered (it introduces a new dimension), the payload efficiency depends on both the the efficiency of parallelization and the number of the processors in the system, as described by Eq. (4) and displayed in Fig. 2. Under the conditions of supercomputing, the expected high performance puts strong constraints both on the number of the processors and the efficiency of their parallelization. As the figure displays, in order to get away from the strongly non-linear part of this 2-dimensional surface (i.e. to have a reasonable efficiency), the present (as of 2018 November) top 5 supercomputers must show up a good bargain between having large single-processor performance and at the same time showing up good effective parallelism.

The figure shows also how the "benchmarking performance (HPL)" of the top 5 supercomputers are positioned on the 2-dimensional surface, while the "real-life performance (HPCG)" values of the efficiency are of two orders of magnitude lower. It is crucial to see that increasing only the number of the cores has a reverse effect on the efficiency of the parallelization, and so leads to a decreasing payload performance, see the right side of Fig. 2. It is interesting to see that the unique HW solution of Taihulight resulting in outstanding parallalization efficiency (especially at that high number of cores) works only when measured with HPL and it draws back when measuring with HPCG. Recall also, that the AI applications are expected to have effciency about 1% (the typical efficiency of HPCG class), and introducing the "biological clock cycle" in brain simulation, decreases the efficiency further by 2-3 orders of magnitude.

### The contribution from the “clock signal”

As discussed shortly in Sect. 3 and in more details in [35], the non-parallelizable fraction of the computing load sums up from different contributions to $(1-\alpha _{\mathrm{eff}})$. In the case of processors, the contribution due to utilizing clock signals is about $10^{-13}$ when using hours of execution times,^4^ and in the case of using 10 s simulation time it is about $10^{-10}$. In the case of brain simulators working on $1\,$ms time grid,^5^ one “neuron operation” is $10^6$ times longer than a “processor operation” (typically $1\,$ns); correspondingly the achievable $(1-\alpha _{\mathrm{eff}})$ contribution would be $10^{-7}$ for supercomputer benchmarking time and $10^{-4}$ for the 10 s simulation time.

This also means that for a neural network simulation task utilizing a $1\,$ms grid time the performance gain cannot be higher than $10^4$; however, the exceptionally high “node performance” may cover this fact. This contribution alone roughly corresponds to the “practical efficiency” of supercomputers, measured by the benchmarks HPCG [38]. The AI applications usually run using shorter floating numbers, typically a $2\cdots 4$ multiplier better payload performance appears by them, but the intensive data exchange between the cores overcompensates this. Consequently, roughly the same performance will be seen for AI applications and HPCG class applications, with the same saturation value and payload performance (see also Fig. 1). To conclude a more accurate value is not possible without making dedicated measurements: utilizing “near” and “far” memories, optimizing thread access [36], using activation functions with different computational need, etc. can considerably change the value. Similarly, the intensive data exchange between the nodes (and threads) of the neural networks is very sensitive to the computing paradigm. The processor deploying “cooperative computing” [29] considerably enhances the computing performance in the case of the HPCG benchmark [26], which computationally is very similar to the AI problems.

### The other contributions

The biggest contribution in the case of brain simulator of this kind comes from context switching. As estimated for the many-thread simulation running on a general-purpose supercomputer [4] “*the computation is split roughly as 10% or 20,000 CPU cycles per time step for neural updates and 90% or 180,000 CPU cycles for synapse processing*”, the frequent context switching takes about 10 % of the total execution time, i.e., the value of $(1-\alpha _{\mathrm{eff}})$ is $10^{-1}$; despite of that the mentioned 20,000 CPU cycles roughly correspond only to one context switch per time step plus several inter-thread switching between neurons. This is why special-purpose HW brain simulator has been built where the “*flagship goal is to be able to simulate the behavior of aggregates of up to a billion neurons in real time*” [39].

The design principle of SpiNNaker [39] is that “*A single SpiNNaker core is a single ARM9 processor ...and is expected to multiplex the behavior of around 1000 neurons*”, it also involves that if all neurons would be used in the same grid time slot, around 2000 context switches would be required for the operation.^6^ To implement this, around $3*10^7$ clock cycles [28] would be required only for context switching, in this way exceeding the available $3*10^6$ clock cycles (assuming $3\,$GHz processors and $1\,$ms grid time slot). The available measured data referring to an ARM processor [40] show that the number of the cycles needed for context switching is in the same order of magnitude as that for Intel processors [28], so the number of neurons must be considerably lower than the theoretical capacity mentioned.

This is why according to [4] “*Each of these cores also simulates 80 units per core*”: when reducing the number of the required context switchings, the core has also some time to make payload calculations. Because of this, it is quite plausible that NEST and SpiNNaker produce comparable performance data under the conditions of the benchmarking by [4]: they by design spend a considerable fraction of their time with the non-parallelizable (and non-payload) activity of making context switches and they both have a hidden unintended $1\,$ms internal clock signal.^7^ Also, this is why “*To obtain an accuracy similar to that of NEST with 0.1 ms time steps, SpiNNaker requires a slowdown factor of around 20 compared to real time*” [4]: otherwise, some of the neurons cannot finish the required calculation until the clock signal arrives. This indirectly underpins that the actual operating parameters are close to their limiting value: the ten times more data traffic (including ten times more in-chip data packets), the ten times more calculations, the ten times more context switches require twenty times more time. In this particular case, all other contributions are of orders of magnitude lower, even when a proper optimization of thread handling considerably increases the effective parallelism [36]; at the same time also demonstrating that different *thread handling affects the performance of the simulation sensitively* and so would do reducing the number of the required context switchings. The final reason is the improperly designed layering of computing: to use operating system services and/or peripherals (like network interfaces) the context switching is inevitable.

Notice that the analysis in the case of supercomputers and neural network simulators shall consider contributions with different weights. In the case of the supercomputers, the $1\,$ns clock period forces to consider also the effect of the physical size and the number of the processors. *In the case of neural network simulators the “clock period” and the context switching are the dominating contributions and (at least at reasonably sized computers) all other contributions can be neglected.*

Also an issue is that the artificial neurons work “at the same time” (biological time) and they are scheduled essentially in a random way on the wall-clock time scale. Since the neurons integrate “*signals from the past*”, this method is not suitable for simulating the effects of spikes with decay time comparable to length of the grid time. In addition, this grid time is “*making all cores likely to send spikes at the same time*” and “[SpiNNaker]does not cope well with all the traffic occurring within a short time window within the time step” [4]. This is why the designers of the HW simulator [39] say that the events arrive “more or less” in the same order as they should. The difference of the timely behavior is also noticed by [4]: “*The spike times from the cortical microcircuit model obtained with different simulation engines can only be compared in a statistical sense*”.

### How performance bound manifests

Recall that the performance limit was derived from the commonly used $1\,$ms integration time and the SPA, both for the SW simulator and the HW simulator. This also explains why they produce a comparable absolute performance: because of utilizing the same integration time as clock period (this enables a neuron to enter the next time station) the parallelization gain is about the same, only the single-processor performance decides about the payload performance. The “single neuron performance” has a special interpretation here: it is the sum of all operations done by a physical core in a biological time slot. So, the single-processor performance is exceptionally high in the case of neural network simulators: the “neuron core” executes instructions in $1\,$ms (for ca. 100 neurons) rather than in $1\,$ns. In these time-slots typically differential equations are solved, context switchings made, waiting executed, so the processing capacity is quite poorly utilized. This is why the efficacy is not increasing as expected. The experience is common for processor scientists [41]: “*we believed that the ever-increasing complexity of superscalar processors would have a negative impact upon their clock rate, eventually leading to a leveling off of the rate of increase in microprocessor performance*”. And, simulating neuron activity on a sequential-parallel digital system is really complex. An indirect proof of the limiting effect of this implicit clock signal is that the analog electronic simulators of neural networks can outperform thousands of times the biological nerve system and the ones based on general-purpose processors.

One must be careful with statements on scalability. According to their goal [4] to implement simulation of a Cortical Microcircuit Model, the authors performed measurements only with a fragment of the available supercomputing capacity (768 cores out of the 458,752) and similarly using 6 (out of the 600 total) SpiNNaker boards; see also the red dot in Fig. 1. At that performance, the nonlinearity occurring at higher performance (see the black dots) cannot be foreseen.

Even in the case of peta-scale neural computing [42], however, this saturation/limitation is the reason of the conclusions like “*the algorithms creating instances of model neurons and their connections scale well for networks of ten thousand neurons, but do not show the same speedup for networks of millions of neurons*” [36] (Fig. 7) and the pessimistic prediction [4] “*Today’s supercomputers require tens of minutes to simulate 1 s of biological time and consume megawatts of power. This means that any studies on processes like plasticity, learning, and development exhibited over hours and days of biological time are outside our reach.*” Notice that [42] has populated the memory of a much larger configuration, but no data like how many neurons were actually used or how many spikes processed are available.

**Fig. 3:**
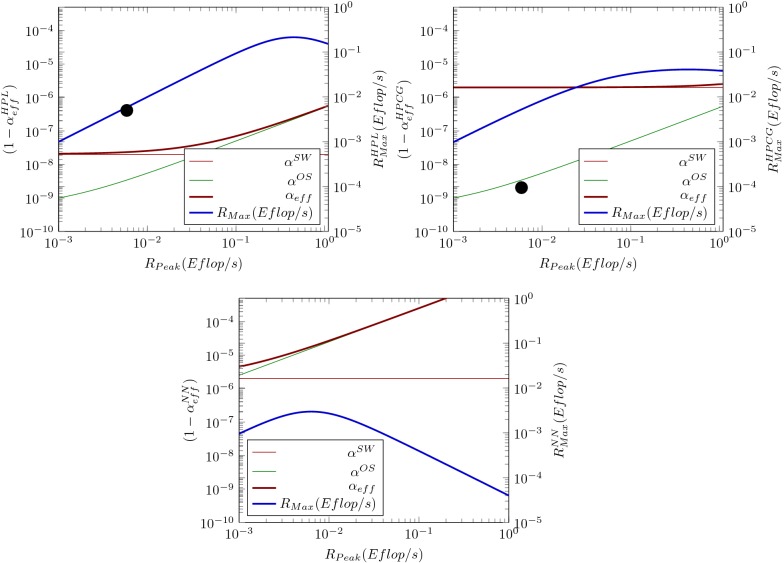
Contributions $(1-\alpha _{\mathrm{eff}}^X)$ to $(1-\alpha _{\mathrm{eff}}^{\mathrm{total}})$ and max payload performance $R_{\mathrm{Max}}$ of a fictive supercomputer ($P=1\,Gflop/s$ @ $1\,$GHz) in function of the nominal performance. The blue diagram line refers to the right-hand scale ($R_{\mathrm{Max}}$ values), all others ($(1-\alpha _{\mathrm{eff}}^{X})$ contributions) to the left scale. The top left figure illustrates the behavior measured with benchmark HPL. The looping contribution becomes remarkable around 0.1 Eflops, and breaks down payload performance when approaching 1 Eflops. The black dot marks the HPL performance of the computer used in works [4, 36]. In the top right, the behavior measured with benchmark HPCG is displayed. In this case, the contribution of the application (thin brown line) is much higher, the looping contribution (thin green line) is the same as above. As a consequence, the achievable payload performance is lower and also the breakdown of the performance is softer. The black dot marks the HPCG performance of the same computer. The bottom figure demonstrates what happens if the clock cycle is 5000 times longer: it causes a drastic decrease in the achievable performance and strongly shifts the performance breakdown toward lower nominal performance values. The figure is purely illustrating the concepts; the displayed numbers are somewhat similar to the real ones

Figure 3 attempts to provide a feeling on the effect of the software contribution and of an increased clock period length. A fictive supercomputer (with behavior somewhat similar to that of supercomputer ${{\it Taihulight}}$) is modeled, and combined with an increased clock period length. All subfigures have dual scaling. The blue diagram line refers to the right-hand scale and shows the payload performance; all the rest to the left-hand scale and display $(1-\alpha _{\mathrm{eff}}^{XX})$ (for the details see [35]) contributions to the non-parallelizable fraction.

Since the behavior of the brain simulation is more similar to the “real-life” applications, the starting point to demonstrate the effect of the considerably increased clock period is the HPCG benchmark (see the top right Fig. 3). As displayed on the bottom figure, the increased clock period results in considerably higher contribution from the operating system (green line), making it dominant. This results in both a considerable decrease in the payload performance and shifts the peak of the payload performance toward lower nominal performance values. Notice that the performance breakdown shown in the figures were experimentally measured by [36] (Fig. 7) and [4] (Fig. 8). Because of the architectural features of the implementations (more neural processors in a computer core, mixing OS scheduling and time-stamped messages, changing contributions due to context switching, etc.), the diagram lines describe the behavior only qualitatively. Notice, however, that the difference in the behavior manifests only at relatively high-performance values (produced with high number of cores).

## How to overcome the issues

The discussion above explains that a huge factor of performance is lost because of using a “time grid” and a wrong computing stack; both are the consequence of the 70-year-old single-processor approach. As the numerical examples above demonstrate, the length of the clock period and the need for context change are in close competition for dominating the performance, but anyhow they together produce a strong upper bound for the performance.

This simple qualitative analysis finds 3 orders of magnitude of the lost performance: the (apparent) performance loss is due to the thousand times shorter execution time: the merit of the parallelism depends on the length of the measurement time. The rest can be attributed to that the simulator makes control and timing, and it takes a considerable amount of time for switching context and making the needed calculations; i.e., that *the present general-purpose processors cannot make a better job when imitating neurons*. As it was demonstrated by [36], through organizing the threads in a more reasonable way, the performance of the simulators can be considerably enhanced. Those solutions, however, only influence the magnitude of the saturation and the position where it occurs: *the saturation is an inherent feature of systems simulating the operation of brain on sequential-parallel systems*. As discussed in [43], the “general-purpose” processors are focussing on computing, rather than on interacting with each other. The importance of the cooperation is well underpinned by the fact that the processor supporting “cooperative computing” [29] kept supercomputer ${\it Taihulight}$ in the first slot on the list of top supercomputers as well as that through optimizing internally the calculations utilizing the cooperation of the processors can considerably enhance the (apparent) computing performance of the supercomputer [26].

When implementing artificial neurons, more than one abstraction levels shall be introduced. At behavioral level, abstract rather than physiological parameters and less computing-intensive methods of calculations should be used. The performance can also be enhanced through introducing direct notifications and core-level (rather than system level) timing, using ideas from [29, 43]. For more details see [35]. The real solution, however, would be to renew the outdated SPA computing model [8] (when utilizing processors for simulating the brain operation). Since the modeled biological objects have no central clock cycle, the other way round would be to design (from scratch) a new architecture, a biologically inspired neural network, rather than adapting system of parallelized processors and components based on the principles of sequential processing.

## Summary

Simulating the inherently massively parallel brain utilizing inherently sequential conventional computing systems is a real challenge. According to the recent studies [4, 36] both the purely SW simulation and the specially designed (but SPA processor-based) HW simulation currently show very similar performance limitations and saturation. The present paper interpreted why even the special-purpose HW simulator cannot match the performance of the human brain in real time. It was explained that the reason is the operating principle itself (or more precisely: the computing paradigm plus its technical implementation together). Based on the experiences with the rigorously controlled database of supercomputer performance data, the performance of the large artificial neural networks was placed on the map of performance of the high-performance computers. The conclusion is that *processor-based brain simulators using the present computing paradigms and technology surely cannot simulate the whole brain (i.e., study processes like plasticity, learning, and development), and especially not in real time*.

## Footnotes

1 Taking this two clock periods as an ideal (but not realistic) case, the actual limitation will surely (much) worse than the one calculated for this idealistic one. The actual number of clock periods depends on many factors, as discussed below.

2 With the present technology the best achievable value is ca. $10^{-6}$, which was successfully enhanced by clustering to ca. $2*10^{-7}$ for ${{ Summit}}$ and ${ Sierra}$, and the special cooperating cores of ${\it  Taihulight}$ enabled to achieve $3*10^{-8}$.

3 https://en.wikipedia.org/wiki/PEZY_Computing: The name PEZY is an acronym derived from the greek derived Metric prefixs Peta, Eta, Zetta, Yotta.

4 Recall that the unrealistic ideal case of utilizing only 2 clock cycles for fork/join operations and a zero-sized computer is assumed.

5 Notice that even SpiNNaker utilizes by design an unintended clock signal: “*An external, self-contained, instantaneous signal drives state change in each process, which contains a trigger that will initiate or alter the process flow”* [37].

6 For the sake of simplicity it is not considered that that same scheduler must do all context switchings. Although [39] emphasizes that “*there is no conventional operating system running on the cores*”, using interrupts and preemptive scheduling make using context switching a must.

7 Again: here the effect of the clock signal and the non-parallelizable contribution arising from the need of context switching are competing for the dominance, and without dedicated measurements there is no way to tell which of them finally dominates.
